# Protocol for designing INVITES-IN, a tool for assessing the internal validity of *in vitro* studies

**DOI:** 10.1080/2833373x.2023.2232415

**Published:** 2023-08-31

**Authors:** Camilla Svendsen, Paul Whaley, Gunn E. Vist, Trine Husøy, Anna Beronius, Emma Di Consiglio, Ingrid Druwe, Thomas Hartung, Vasiliki I. Hatzi, Sebastian Hoffmann, Carlijn R. Hooijmans, Kyriaki Machera, Joshua F. Robinson, Erwin Roggen, Andrew A. Rooney, Nicolas Roth, Eliana Spilioti, Anastasia Spyropoulou, Olga Tcheremenskaia, Emanuela Testai, Mathieu Vinken, Gro H. Mathisen

**Affiliations:** a Norwegian Scientific Committee for Food and Environment, Norwegian Institute of Public Health, Oslo, Norway; b Department of Chemical Toxicology, Norwegian Institute of Public Health, Oslo, Norway; c Lancaster Environment Centre, Lancaster University, Lancaster, UK; d Division for Health Services, Norwegian Institute of Public Health, Oslo, Norway; e Department of Food Safety, Norwegian Institute of Public Health, Oslo, Norway; f Institute of Environmental Medicine, Karolinska Institutet, Stockholm, Sweden; g Environment & Health Department, Italian National Institute of Health (ISS), Rome, Italy; h United States Environmental Protection Agency, Office of Research and Development, Center for Public Health and Environmental Assessments, Research Triangle Park, NC, USA; i Center for Alternatives to Animal Testing (CAAT), Johns Hopkins University, Bloomberg School of Public Health, Baltimore, MD, USA; j CAAT Europe, University of Konstanz, Konstanz, Germany; k Laboratory of Toxicological Control of Pesticides, Scientific Directorate of Pesticides’ Control and Phytopharmacy, Benaki Phytopathological Institute, Kifissia, Greece; l Evidence-Based Toxicology Collaboration (EBTC), Johns Hopkins University, Bloomberg School of Public Health, Baltimore, MD, USA; m SEH consulting + services, Paderborn, Germany; n Department of Anesthesiology, Pain and Palliative Care, Radboud University Medical Centre, Nijmegen, Netherlands; o Department of Obstetrics, Gynecology & Reproductive Sciences, University of California, San Francisco (UCSF), CA, USA; p 3Rs Management and Consulting ApS, Lyngby, Denmark; q Division of Translational Toxicology, National Institute of Environmental Health Sciences, Research Triangle Park, NC, USA; r Department of Pharmaceutical Sciences, University of Basel, Basel, Switzerland; s Swiss Centre for Applied Human Toxicology (SCAHT), Basel, Switzerland; t Department of Pharmaceutical and Pharmacological Sciences, Vrije Universiteit Brussel, Brussel, Belgium

**Keywords:** Cell culture, NAMs, next generation risk assessment, risk of bias

## Abstract

This protocol describes the design and development of a tool for evaluation of the internal validity of *in vitro* studies, which is needed to include the data as evidence in systematic reviews and chemical risk assessments. The tool will be designed specifically to be applied to cell culture studies, including, but not restricted to, studies meeting the new approach methodology (NAM) definition. The tool is called INVITES-IN (IN VITro Experimental Studies INternal validity).

In this protocol, three of the four studies that will be performed to create the release version of INVITES-IN are described. In the first study, evaluation of existing assessment tools will be combined with focus group discussions to identify how characteristics of the design or conduct of an *in vitro* study can affect its internal validity. Bias domains and items considered to be of relevance for *in vitro* studies will be identified. In the second study, group agreement on internal validity domains and items of importance for *in vitro* studies will be identified via a modified Delphi methodology. In the third study, the draft version of the tool will be created, based on the data on relevance and importance of bias domains and items collected in Studies 1 and 2. A separate protocol will be prepared for the fourth study, which includes the user testing and validation of the tool, and collection of users’ experience.

## 1. Introduction

### 1.1. Evaluation of internal validity

This protocol describes the design and development of a tool for evaluation of the internal validity of *in vitro* studies. Internal validity is the extent to which a study (methodological design, methods and data analysis) is free from bias, where bias is ‘systematic error, or deviation from the truth, in results’ (Cochrane Collaboration 2005). A test performed *in vitro* (‘in the glass’) means that it is done outside of a living organism and it usually involves isolated tissues, organs or cells (ECHA 2023). The tool is called INVITES-IN (IN VITro Experimental Studies INternal validity).

Methods to generate evidence for regulatory toxicology are shifting from classical animal experiments to new approach methodologies (NAMs). The European Chemicals Agency and the U.S. Environmental Protection Agency define NAMs as any technology, methodology, approach or combination that can provide information on chemical hazard and risk assessment without the use of animals, including *in silico*, *in chemico*, *in vitro* and *ex vivo* approaches (ECHA 2016; EPA 2018). According to the European Food Safety Authority (EFSA), the term NAMs is used to make reference to any non-animal-based approach that can be used to provide toxicological information in the context of hazard/risk assessments (EFSA et al. 2022).

As part of the gradual incorporation and transition toward the use of NAMs, including *in vitro* studies, a framework for evidence-based use of NAMs in toxicological research and chemical risk assessment is required. Such a framework should ultimately incorporate at least the following principles:

1. Result in identification of all relevant NAM-generated evidence relating to the research question addressed in a systematic review or risk assessment.
2. Provide for the evaluation of the internal validity of NAM studies (propensity for systematic error due to how the study is designed and conducted).
3. Provide for the evaluation of the external validity of NAM studies (the degree to which results of a study can be translated/generalised to human adverse health effects).
4. Contribute to objectivity, robustness, transparency and reproducibility in the hazard identification and characterisation process.
5. In its approach to normalising and structuring the description and analysis of NAMs, contribute to progress in the extent to which research data conform to FAIR (Findable, Accessible, Interoperable and Re-usable) principles of open science.

Systematic review and evidence-based toxicology principles should be implemented in all parts of the framework, and it should be generic and usable across different regulatory sectors such as food safety, cosmetic ingredient safety, etc. Principles for incorporating evidence from NAMs into risk assessments and a framework for the evaluation of skin sensitisation have been developed for cosmetic ingredients (Dent et al. 2018; Gilmour et al. 2020). Methods for incorporation of mechanistic studies as supporting evidence in hazard and/or risk assessment is included in the U.S. NTP OHAT handbook for systematic reviews, the ORD staff handbook for developing IRIS assessments, and the draft TSCA interpretation of systematic review methods to support chemical risk evaluations (EPA 2022, 2023; NTP OHAT 2019). However, there is currently no complete framework for evidence-based chemical risk assessment that integrates NAMs to facilitate the transition from use of animals to the use of NAMs in chemical risk assessments.

‘Next generation risk assessment in practice’ is a project in the European Partnership for the Assessment of Risks from Chemicals (PARC). PARC aims to develop next generation chemical risk assessment to advance research, share knowledge and improve skills, protecting human health and the environment. The present project is included in the task focusing on facilitating regulatory acceptance and use of NAMs. PARC is a 7-year partnership under Horizon Europe, including close to 200 institutions from 28 countries working in the areas of the environment or public health, and 3 EU authorities (PARC 2023). With the ‘Next generation risk assessment in practice’ project, we aim to contribute to the development of a framework for evidence-based use of data generated by *in vitro* studies in human health hazard identification and characterisation by creating tools and guidances. A webpage giving an overview of the planned work in the ‘Next generation risk assessment in practice’ project has been created (VKM 2023). The first step in our PARC project is to develop a tool for evaluation of internal validity for *in vitro* studies. The next steps, all focusing on *in vitro* studies, will be the development of a tool for evaluation of external validity, creation of guidance for evaluation of certainty in the evidence, and creation of guidance for the identification of point of departure and the uncertainty in the point of departure. We chose to start focusing on creation of tools for validity assessment, as validity assessment is one of the critical steps in the systematic review process. Further, we chose to start focusing on *in vitro* models as there is a general agreement that these are important as replacement for animal studies to provide information for hazard/risk assessment (ECHA 2016; EFSA et al. 2022; EPA 2018) in a wider integrating approach. It has been suggested that *in vitro* models could be more suitable than animal models for the prediction of toxicity. For example, *in vitro* data did predict liver toxicity caused by the drug troglitazone whereas neither published animal nor human studies were able to accurately predict the hazard (Dirven et al. 2021).

Several *in vitro* study designs exist; however, we have chosen only to focus on cell culture studies (meaning studies using cells derived from multicellular organisms). This delimitation is mainly due to feasibility, especially concerning the user testing, where the number of user testing participants will have to be very large to be able to test that the tool works on all types of *in vitro* study designs.

The implementation of this tool might be of help to improve the inclusion of NAMs in the chemical risk assessment process and facilitate regulatory uptake, with a focus on risk assessors’ daily practice and workflow.

While many tools have been created for assessing *in vitro* studies, there is a priori lack of consensus on developing a tool with the application of rigorous methods. We therefore aim to address this situation by using methods that ensure we are building on prior work, with a degree of rigor consistent with our intent to provide an authoritative assessment tool. We also intend to use the findings of INVITES-IN to prepare guidance on the design and conduct of *in vitro* studies that will help researchers minimise and/or transparently identify potential biases in their studies.

### 1.2. Objective

The aim of this project is to create INVITES-IN, a tool for evaluating the internal validity of *in vitro* studies. The INVITES-IN tool will be designed specifically to be applied to cell culture models (e.g., cell lines, primary cell models, co-cultures, monolayer and 3-D cell models systems) treated with a single-chemical substance exposure, measuring any outcome. We anticipate that the tool will be applicable (potentially with modification) to other *in vitro* study designs or other NAMs such as organ-on-a-chip, *in ovo, fish embryos, ex vivo*, *in chemico,* etc., and chemical mixture studies, but this will not be addressed in this study.

To contribute to its usability, INVITES-IN will be accompanied by instructions to guide the user through the evaluation of internal validity of *in vitro* studies step-by-step. While there is good empirical evidence from several domains that certain features of how a study is designed, conducted and analysed can introduce bias, it is usually not possible to determine how much bias a given feature has introduced on any specific occasion (Savović et al. 2012). INVITES-IN therefore follows conventional guidance (Boutron et al. 2022; Frampton et al. 2022) in being designed to differentiate studies with relatively higher risk of bias from studies with relatively lower risk of bias.

### 1.3. Project governance

The development of INVITES-IN is part of the PARC project ‘Next generation risk assessment in practice’ [Project 101057014 – PARC]. A project group (PG) has been established with the responsibility for developing and implementing the tool for evaluation of internal validity of *in vitro* studies. The project is led by the Norwegian Institute of Public Health represented by the Norwegian Scientific Committee for Food and Environment (Norway). The project partners are Benaki Phytopathological Institute (Greece), Istituto Superiore di Sanità (Italy) and the University of Basel (Switzerland).

A scientific advisory group (SAG) consisting of experts in systematic review principles, chemical risk assessment, toxicology, NAMs and/or methods for tool development, several of whom have been directly involved in developing approaches to assessing the validity of *in vitro* studies, has been established. The SAG gives strategic guidance and support to the PG and share information about ongoing projects addressing similar questions to ensure that the outcome of this project complements and builds on the work of others and thereby creates synergies and avoids duplication of efforts.

## 2. Materials and methods

### 2.1. Study design

#### 2.1.1. An overview of the creation of INVITES-IN

**Figure 1.**
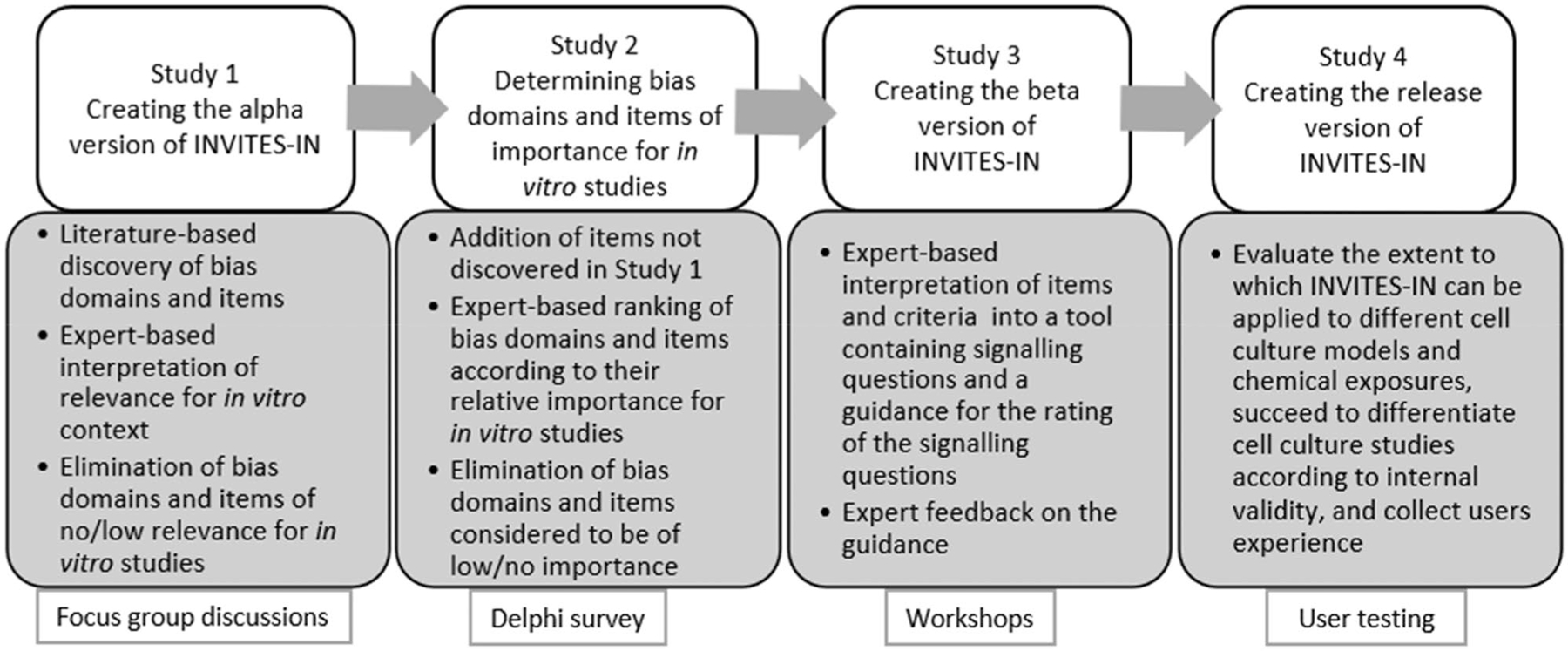
An overview of the four studies that will be performed to create the release version of INVITES-IN.

**Figure 2.**
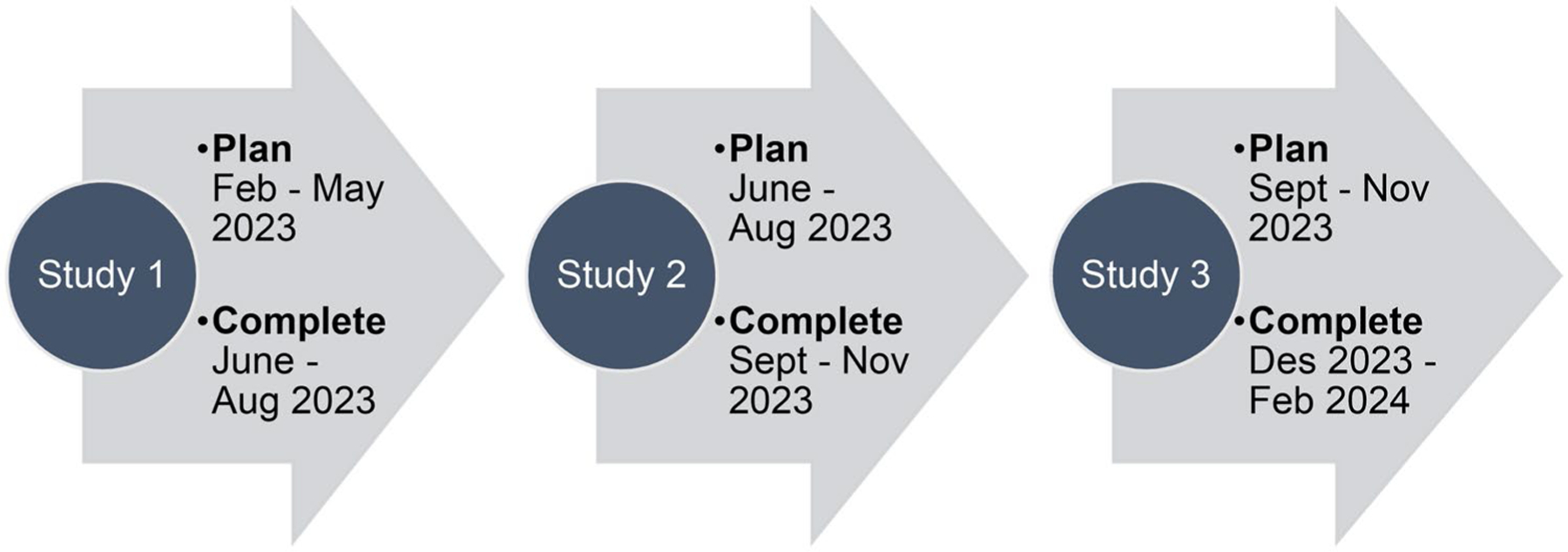
An overview of the 2023–2024 timeline for the creation of the beta version of INVITES-IN.

The method for creating INVITES-IN will follow the general framework for developing quality assessment tools suggested by Whiting et al. (2017). This is a broad framework of general principles rather than a tightly prescribed standard but gives the general structure of our approach. Four studies will be performed to create INVITES-IN (Figure 1). This protocol describes Studies 1, 2 and 3, and the timeline is shown in Figure 2. A separate protocol will be prepared for Study 4.

The tool will consist of signalling questions and criteria for reaching risk-of-bias judgments for each signalling question. Criteria are the issues that have to be fulfilled to avoid bias. Signalling questions are questions that the users of the tool answer in order to determine whether the criteria have been fulfilled. The technical solution for the tool has not yet been decided; however, we intend to make an online tool.

The target group for the use of the tool (i.e., end-users) includes *in vitro* scientists and risk assessors conducting literature reviews in hazard assessments/safety evaluations, which could be part of a chemical risk/safety assessment, a systematic review or both, for regulatory or research purposes.

To get the input we need to develop the tool, we aim to recruit participants experienced with *in vitro* research that are representative for the end-users. For the Studies 1 and 3, we aim to recruit some participants also having experience with systematic reviews, some also having experience with chemical risk assessment, and some having no experience with systematic reviews or chemical risk assessments. For Study 2, we consider it critical that all participants have systematic review experience, as this is the study where the importance of different internal validity items will be ranked. Previous experience with evaluation of internal validity is considered important to be able to rank importance of different internal validity items. All groups of end-expected users are covered by the networks of the PG and the SAG. Potential participants will therefore be identified through nomination by PG and SAG members, who will be requested to nominate three potential participants. For each nominated participant, an overview of their scientific expertise and experience, affiliation, geographical location and gender will be prepared. From the pool of nominated participants, PG will select participants that will be invited. In the selection process, PG will ensure diversity among the participants by including scientists from different fields having different professional backgrounds and experience with different cell culture models, covering a variety of geographical locations, and having an even gender distribution. In each focus group, all participants should be affiliated with different institutions, located in at least four different countries. This way we will avoid having an overrepresentation of focus group participants from a few institutions or from a too limited number of countries. We consider that this described process will make it possible to carry out the recruitment without it being an overly time-consuming process, and at the same time secure sufficient diversity in the group of participants.

**Figure 3.**
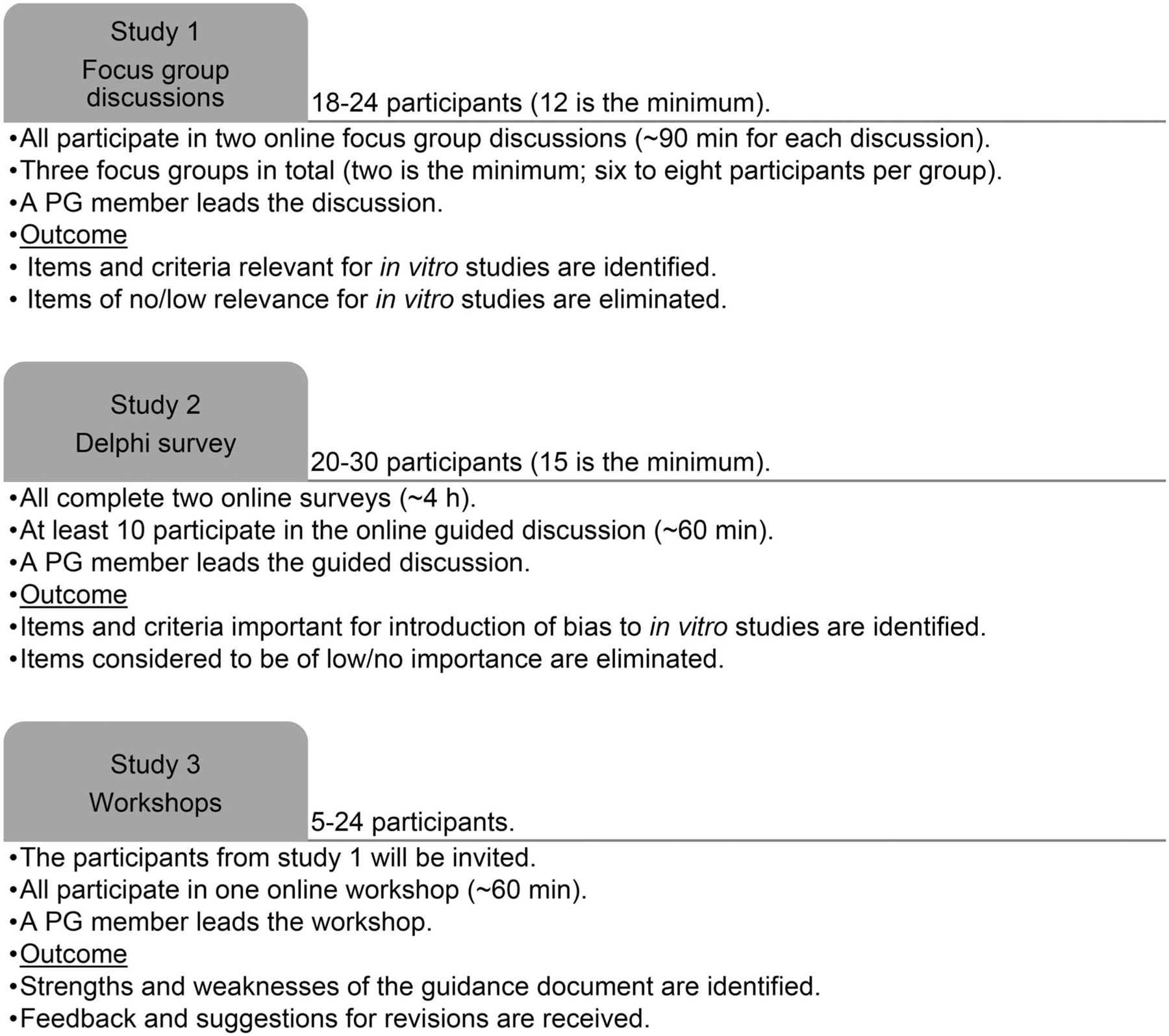
Participants’ tasks and workload in Studies 1–3, and the outcome of their contribution.

The tasks and workload for the participants, the outcome of their contribution and the participant eligibility criteria, are shown in Figure 3 and Table 1. Note that it is not expected that the same persons participate in all studies. It is planned that the persons participating in Study 1 will be also invited to participate in Study 3.

For all three studies, the potential participants will receive information about the project when they are contacted by email, and participants that accept the invitation will be requested to complete a declaration of interest form. The PG will evaluate the declaration of interest forms, focusing mainly on identification of potential conflicts of interest that may interfere with the participants’ contribution and role in the focus group discussion.

Previous studies report average or median time for the assessment of RoB of a study to range from 20 to 40 min (Eick et al. 2020; Momen et al. 2022). We intend to keep the time needed for assessment of one cell culture study within this range.

All data analyses will be done by the PG members. All raw data from each study will be anonymised and made available as supplementary to the respective publications.

#### 2.1.2. Ethical review

Ethical approval has been given by the Norwegian Institute of Public Health.

### 2.2. Study 1: Creating the alpha version of the tool

#### 2.2.1. Introduction and objective

The objective of Study 1 is to create a straw-man or alpha version of INVITES-IN that can be further developed via a modified Delphi process (see Section 2.3.2 for description). In Study 1, a list of characteristics of the design, conduct and analysis of an *in vitro* study that can introduce bias into its results or findings will be compiled, organised thematically and then interpreted into a draft set of structured signalling questions that constitute the alpha version of INVITES-IN.

The knowledge goal is to have the expert interpretations of the relevance of bias domains and items for *in vitro* studies.

A pilot focus group discussion was arranged to get an impression of the time needed for the focus group discussions, to test the technical functions and to get feedback on factors related to the presentation of questions and the use of examples that may be of importance to conduct successful focus group discussions.

#### 2.2.2. Method

We will include three focus groups with six to eight participants in each group (Figure 3).

An overview of the workflow and the responsibilities in Study 1 are given in Table 2.

##### 2.2.2.1. Identifying relevant bias domains and items.

A list of bias domains and items of potential relevance for *in vitro* studies will be prepared using several literature sources. This list will serve as a starting point for the creation of INVITES-IN and provide the basis for the focus group discussions. The literature sources are as follows: two systematic reviews on validity tools for *in vitro* models (Tran et al. 2021; Whaley, Hooijmans, and Wattam in preparation), a publication on study sensitivity that includes assessment items that may relate to internal validity but may not be included in other tools (Cooper et al. 2016) and tools for evaluation of risk of bias (EPA 2022; NTP OHAT 2015, 2019; Roth, Zilliacus, and Beronius 2021; Sterne et al. 2019).

##### 2.2.2.2. Focus group participants.

Eligible focus group participants will be scientists with or without systematic review experience that are active in the field of *in vitro* research in academia, governmental institutions (including risk assessment institutions and research institutes) or private research institutes, at post-doctoral level or higher, and level B1 English speakers (Table 1). PG and SAG will nominate participants. We aim to have an equal gender distribution, a reasonable demographic and regional distribution, and a group size of six to eight participants as this group size is recommended to generate diverse ideas but not so many participants that they do not have a chance to share perspectives (Krueger et al. 2001). The minimum number of participants in a focus group is considered to be four. All participants in a focus group will be affiliated with different institutions in an attempt to achieve variation in input and perspective, and they should be working with a variety of *in vitro* models to cover a wide range of experimental systems. No compensation is offered for the participation, and participants will not be offered co-authorship.

Potential focus group participants will be contacted via email. They will receive a document with information about the project, the purpose of the focus groups and the focus group discussions, that the use of information learned in the meeting will not allow for identification of the focus group participants, the withdrawal procedure, the financial source and the approximate time for the focus group meeting. Focus group participants must actively confirm their consent by email.

We aim to have three different focus groups (Krueger et al. 2001); however, two groups are considered to be the minimum. All groups will be presented with the same information and questions, although the direction in which discussion is steered may depend on how comprehensively previous focus groups were able to cover each issue. The need for including an additional group will be discussed if new insights are presented during the meetings, or if areas needing discussion were not addressed.

##### 2.2.2.3. Focus group discussion.

We plan to have two group discussions per focus group. The second meeting will be cancelled if considered not to be needed. The discussions will be carried out as online meetings and will be recorded. A PG member will act as a focus group moderator and lead the discussions in the meeting, and another PG member will handle the logistics (the assistant moderator).

The complete list of identified bias domains and items will be the starting point for the focus group discussions. The discussions will be facilitated with a view to addressing two questions (numbering is for referencing purposes and the questions will not necessarily be presented in this order):

1. Are there any gaps in the identified domains or items that could influence systematic error in an *in vitro* study?
2. What characteristics of the design, conduct or analysis of an *in vitro* study could introduce systematic error into its results or findings?

Question (1) will be addressed both by asking directly and inferred from analysis of the discussion (see Section 2.2.2.4). Question (2) will be directly asked.

Discussion relating to questions (1) and (2) will be structured in terms of the bias domains defined in the Scientific Evidence Code System (SEVCO) (Table 3) (Alper et al. 2021b). The SEVCO domains are chosen because they are consistent with the bias domains of Whaley, Hooijmans, and Wattam (in preparation) and the OHAT tool (NTP OHAT 2019) but represent a more recent normalised list of bias categories derived from a robust grounding and consensus process (Alper et al. 2021a). These definitions are developed for human studies, and the relevance for *in vitro* studies will be discussed in the focus groups. We acknowledge that not all bias domains presented in Table 3 may be of relevance for *in vitro* studies. However, we will include all bias domains with approved SEVCO definitions in the focus group discussions in order to collect expert feedback on the relevance for *in vitro* studies. SEVCO draft bias domains that have not been approved are not listed. Participants may suggest additional bias domains.

Focus group participants will be shown and have read to them the definitions for each bias domain. Participants will then be led in discussion of how the domain might be active in the *in vitro* context, with examples from their practical research experience of how systematic error can be introduced into an *in vitro* study. For each bias domain, one example for animal studies and one example for *in vitro* studies will be prepared, and these will be presented when there is a need for further clarification to start the discussion.

Participants will be given an option to send additional thoughts and considerations on the relevance of the discussed bias domains and items for *in vitro* studies to the PG by email within a week after the focus group discussion.

##### 2.2.2.4. Data analysis and reporting.

Focus group transcripts will be analysed for potential risk of bias criteria and items that could be added to the alpha version of INVITES-IN. For time efficiency, transcripts of the focus group discussions will be machine generated. Errors in transcription will only be corrected when they affect coding and interpretation of the discussion and will be done by the focus group moderator and the assistant moderator. Anonymised transcripts will be shared as raw data and be included as supplementary materials. The original recordings, as they contain personally identifiable information, will not be made available.

The focus group transcripts will be annotated (coded) in order to provide qualitative data on the following: preferences of the participants for traditional versus more recent approaches to structure risk of bias assessment (‘preferred approach’), including reasons for and against; the participants’ ideas about how researchers’ approaches to designing, conducting, analysing and reporting studies (‘issues’) can potentially introduce systematic error, including their potential importance; the participants’ ideas about when (‘time-point’) systematic error is introduced; the participants’ ideas about the relevance (‘relevance’) for *in vitro* studies.

Data on preferred approach, issues, time-points and relevance will be annotated by two investigators with a high level of expertise in bias assessment working independently then reconciling their coding decisions in discussion with a third investigator with experience in coding and reconciliation. The annotation environment will be Microsoft Word. The annotators will reach consensus for coding using the codebook through coding a part of one transcript together and discussing differences in interpretation, and they will agree on the rules for annotation (e.g., sentence or word highlighting for codes) and document these as their coding strategy in a coding manual.

Coding will be a mix of deductive (prespecified) and inductive (ad hoc) annotation. The definitions of the deductive codes are included in Table 4, and we have also indicated where we already anticipate that codes will be developed inductively, though further inductive codes will be developed as needed. The Code Book is shown in Table 5. A report of the results of the annotation exercise, as a set of excerpted text strings aggregated under code categories and labelled with specific codes, will be generated as data for supporting development of the alpha version of INVITES-IN.

#### 2.2.3. Results and outcome

The focus group participants will not make decisions but provide ideas and recommendations. Their feedback on issues, time-points, and relevance for the *in vitro* context will be used by the PG to prepare the alpha version of INVITES-IN, which will contain all bias domains and items considered to be of relevance for *in vitro* studies with reasonings. The final decisions regarding the inclusion of bias domains and items in the alpha version of INVITES-IN will be made by the PG members involved in this study. An overview of bias domains and items that are not included in the alpha version will be included in the study report and comprehensively documented in supporting data. The intent here is not to permanently exclude any items, but to generate a list of practical length for analysis by the modified Delphi process. Decisions about exclusion of domains or items at this stage affect only the alpha version of INVITES-IN and are not final: if the Delphi process reintroduces any excluded concepts, this will supersede the initial decision made by the PG.

### 2.3. Study 2: Determining bias domains and items of importance for in vitro studies

#### 2.3.1. Introduction and objective

The objective is to eliminate, add to or refine the proposed bias domains and assessment items that are generated by Study 1. This provides the final data to be interpreted into the beta version of INVITES-IN in Study 3.

The feature tested is the importance of the bias domains and items included in the alpha version of INVITES-IN for the internal validity of *in vitro* studies.

The knowledge goal is to have the expert interpretations of the importance of bias domains and items for *in vitro* studies.

#### 2.3.2. Method

A modification of the Delphi technique (Dalkey and Helmer 1963) will be used to obtain subjective opinions on the importance of bias domains and items for *in vitro* studies from experts experienced with both *in vitro* studies and systematic review principles. The Delphi technique gives the opportunity to collect subjective expert statements anonymously and gives the desired transparency, without e.g. social or personality-based factors resulting in one expert’s feedback influencing the feedback another expert in the group. Therefore, this approach is considered to be an appropriate technique to identify expert agreement.

A two-round digital Delphi survey will be conducted, followed by an online workshop for guided discussions. In both rounds, expert panellists will complete a questionnaire. From each Delphi round, the outcome will be subjective expert feedback on importance of bias domains and items, and we will use these data to identify expert agreement on bias domains and items important for internal validity of *in vitro* studies. Bias domains and items for which agreement was not reached during the two Delphi rounds will be discussed in the workshop. In addition, the participants will be asked to give input on the wording of the questions in each Delphi round and during the guided discussion.

An overview of the workflow and the responsibilities in Study 2 is given in Table 6.

##### 2.3.2.1. Delphi participants.

Eligible Delphi participants will be scientists that are active in the field of *in vitro* research and have some experience with systematic literature review principles, are affiliated in academia, governmental institutions (including risk assessment institutions and research institutes) or private research institutes, at post-doctoral level or higher and level B1 English speakers (Table 1). PG and SAG will nominate participants.

We aim to have an even gender and geographical location distribution for the potential participants that are invited to participate. The number of participants will be 20–30 (see Figure 3), depending on the number of suitable candidates identified by PG and SAG and the candidate’s willingness to participate. The minimum number of participants is considered to be 15.

Potential participants will be contacted via email, and they will receive a letter with information about the project and the purpose of the Delphi survey including the fact that the use of individual survey responses will not allow for identification of the participant, the withdrawal procedure, the financial source, as well as the approximate time for completion of the questionnaires. Participants must actively confirm their consent by email to be included as a participant. Before each Delphi round and the guided discussion, participants will receive instructions. Participants are eligible to be co-authors of the Delphi study manuscript if they also read and comment on the final draft. No compensation or other incentives are offered for the participation.

##### 2.3.2.2. Delphi rounds and workshop with guided discussion.

A Delphi round is defined as the process where the expert panellists complete a questionnaire. Before each round, expert panellists will receive a document with information about the project, the Delphi survey, and how the Delphi questionnaire information will be handled and used.

The PG develops the questionnaire based on the alpha version of INVITES-IN prepared in Study 1. The questionnaire will be prepared as an Excel form, and it will be sent to the expert panellists by email. The expert panellists rate the importance of different bias domains and items for the internal validity of *in vitro* studies. A 5-point Likert scale, with the categories strongly disagree (1), moderately disagree (2), neutral (3), moderately agree (4) and strongly agree (5) is used as response options (Verhagen et al. 1998).

The expert panellists will have two weeks to complete the questionnaire in each Delphi round, and they will receive up to three email reminders to complete each round. Panellists not responding within the deadline in one of the two Delphi rounds will be excluded from that round. Removed participants will not be replaced. Participants excluded from the first round will also be excluded from the second round.

###### Delphi round 1:

The questionnaire is completed by the expert panellists, and they will also be able to suggest additional bias domains and items and alternative wording.

####### Between Delphi rounds 1 and 2:

- The results are analysed, and expert panellists receive feedback on average rating and distribution of ratings of importance of bias domain and items.
- The questionnaire is revised. Bias domains and items that met criteria for identification of agreement for inclusion or exclusion from INVITES-IN are removed. New questions may be included, and existing questions may be revised.

####### Delphi round 2:

The revised questionnaire is completed by the expert panellists.

####### Between Delphi round 2 and the workshop:

- Results are analysed, and expert panellists receive feedback on average rating and distribution of ratings of importance of bias domain and items.
- Bias domains and items that did not reach agreement for either inclusion or exclusion in round 2are included in the guided discussion workshop. An overview of all bias domains and items that did not reach agreement for either inclusion or exclusion will be prepared and sent to the expert panellists who will be requested to include arguments for considering the items to be of higher or lower importance. PG will prepare an overview of all arguments, which will be sent to workshop participants.

####### Workshop:

A workshop will be arranged to have a guided discussion on items where no agreement on importance for *in vitro* studies has been identified. The starting point for the discussion of each of these items will be the overview of arguments created between the Delphi round 2 and the workshop. During the discussion, we will ask the participants to give reasonings for agreeing or disagreeing with the arguments. New arguments that emerge from the guided discussion will be included in the overview. A PG member will lead and moderate the guided discussion. The workshop will be recorded and transcripts from the workshop will form the basis for the revision of the list of arguments.

##### 2.3.2.3. Data analysis and reporting.

One PG member will send out the questionnaires, receive the completed questionnaires from the expert panellists and anonymise the answers. This person will not be involved in the data analysis.

Expert panellist characteristics such as gender distribution and geographic localisation will be reported. The response rate (percentage) for expert panellist completing the Delphi survey will be calculated and reported. The average group response, changes in rating between rounds, as well as modifications of the questionnaire, will be reported. The expert panellists rating of the questions will be analysed independently for round 1, round 2, and the guided discussion, and median, mean, standard deviation and the interquartile range will be reported.

Criteria for identification of agreement in rounds 1 and 2:

- Agreement for inclusion of bias domains and items is identified when 70% of the expert panellists rate the relevance and wording of a question as the category ‘moderately agree’ or ‘strongly agree’ (1 and 2 on the 5-point Likert scale).
- Agreement for exclusion of bias domains and items is identified when 70% of the expert panellists rate the relevance of a question as the category ‘moderately disagree’ or ‘strongly disagree’ (1 and 2 on the 5-point Likert scale).

Decisions on identification of agreement will be made by the PG members involved in this study.

The transcripts from the workshop will be anonymised and made available as supplementary materials.

#### 2.3.3. Results and outcome

Study 2 will result in a list of bias domains and items (i) for which there were agreement that the domain or item is of importance when evaluating risk of bias of *in vitro* studies, (ii) for which there was agreement that the domain or item is not of importance when evaluating risk of bias of *in vitro* studies and (iii) where agreement was not reached for either inclusion or exclusion in the two rounds of Delphi or in the guided discussion. For the items where agreement was not reached, arguments for considering a given item as higher or lower importance will be included.

### 2.4. Study 3: Creating the beta version of INVITES-IN

#### 2.4.1. Introduction and objective

The objective is to create the beta version of INVITES-IN, which will be advanced to user testing. This will consist of two elements: the tool itself, consisting of a set of signalling questions and a process for deriving a risk of bias assessment, and a guidance document explaining how to use the tool. The guidance document will also include relevant examples of ratings of cell culture studies. This will be given as short texts illustrating possible reporting in a publication together with explanations and reasonings for how this is intended to be rated when applying INVITES-IN.

The knowledge goal is to have a complete set of signalling questions addressing bias domains and items of importance for introduction of bias to *in vitro* studies and the criteria for the rating of the questions.

#### 2.4.2. Method

An overview of the workflow and the responsibilities in Study 3 is shown in Table 7.

##### 2.4.2.1. Draft version of INVITES-IN.

The draft version of INVITES-IN will be prepared by the PG. The outcome of Study 2 will be used to formulate the signalling questions. The guidance document will contain explanations of how each signalling question should be rated.

##### 2.4.2.2. Workshop participants.

Members of the focus group participating in Study 1 will be invited to participate in an online workshop, except for those who also participated in the Delphi process which will be excluded. No compensation is offered for participation, and participants will not be offered co-authorship.

##### 2.4.2.3. Workshops.

One or more online workshops will be arranged to collect feedback on both the presentation and the information in the guidance document. Regarding the feedback on information in the guidance document, the focus will be on the suggested criteria for the rating of the signalling questions and whether we have succeeded in formulating these so that it is the factors that are considered to be of greatest importance for the introduction of bias that are given the most weight.

We also attempt to collect feedback from the participants regarding the presentation of the signalling questions from the workshops, whether they should be structured according to the relevant bias domains or be based on study characteristics and structured around whether the bias is introduced before, during or after the exposure of the experimental system to the test item (i.e., prior, during and after the administration of the chemical substance in the experiment).

When possible, the number of participants in a workshop will be six to eight. However, workshops with fewer participants will be considered in order to facilitate participant recruitment. The workshops will be recorded.

##### 2.4.2.4. Data analysis and reporting.

Transcripts of the feedback on the guidance document received in the workshops will be prepared and made available as supplementary materials. Based on the feedback from participants in the workshops, PG will make the final decision on the need for revision.

#### 2.4.3. Results and outcome

The beta version of the tool is ready for user testing.

## 3. Discussion

This protocol describes the methodological approach for the development of the INVITES-IN tool. In this protocol, we have proposed an approach similar to that of ROB2 (Sterne et al. 2019) and ROBINS (Sterne et al. 2016). The approach chosen fulfils the framework for developing quality assessment tools (Whiting et al. 2017), which is to our knowledge the only existing framework for how to develop quality appraisal tools. Although we cannot be certain that the chosen approach is the best approach, we feel confident that the methods chosen are rigorous and have been agreed upon of more than 20 experienced experts/scientists. Also we have focused on transparency, and their detailed method descriptions and collected data (transcripts and more) will be made publicly available. Our methodological approach comprises four separate studies and involves both focus groups, two-round Delphi survey and user-testing at different stages. A separate protocol will be prepared for the user testing (Study 4). Involving groups of experts in every study reduces the level of expert judgements made by the PG and also ensures that the tool development is based on a wide range of feedback from experts that are the intended users of the tool. It might be that including more participants in the three studies described in this protocol would give additional interpretations of the relevance and importance of bias domains and items for *in vitro* studies. It may be a challenge to recruit enough experts to ensure sufficiently powering of the studies. To facilitate the recruitment process, the workload for the participants is limited to the absolute minimum. Also, participants in the Delphi-survey, which is likely to have the largest workload for the participants, will be offered authorship on the Delphi study manuscript.

The described approach will not include the assessment of magnitude or direction of the bias. We believe that these issues need to be addressed by empirical research in addition to expert knowledge elicitation. We acknowledge the importance of assessing magnitude and the direction of bias; however, the amount of work and time it will take to properly address this will not be possible at this stage of the tool development.

Given that assessment of *in vitro* studies is likely to become a fast-moving field, we acknowledge that there may be a need for the tool to be updated to reflect rapid changes in consensus on how to do this, and/or it may be a fast movement towards modifying INVITES-IN for other specific NAM study designs. A plan for the update or modification of INVITES-IN is not included in this protocol, as it is restricted to describe the process for the creation of this tool.

## Figures and Tables

**Table 1.** An overview of the criteria for participation in Studies 1–3.

	Selection of participants	Study 1	Study 2	Study 3
Scientific experience and expertise	*In vitro* models	x		x
	*In vitro* models AND chemical risk assessment	x		x
	*In vitro* models AND systematic review methods	x	x	x
	*In vitro* models AND experienced with the development of relevant guidance documents for chemical risk assessors	x		x
Balancing factors	Academia	x	x	x
	Governmental institutions (including risk assessment institutions and research institutes)	x	x	x
	Private sector research institutions	x	x	x
	Gender distribution	x	x	x
	Demographic distribution	x	x	x
	Regional distribution	x	x	x
Academic level	Post-doctoral level or higher	x	x	x
Language	English, level B1 or higher	x	x	x

**Table 2.** An overview of Study 1.

Phase	Task	Responsible
Plan	Prepare the list of bias domains and items. Create questions for the focus group discussions.	Project group
	Define inclusion criteria for focus group participants. Nominate and recruit focus group participants fulfilling the inclusion criteria.	Project group and scientific advisory group
Actions	Carry out the focus group discussions. Analyse results and prepare the final report.	Project group
Result	Bias domains and items of relevance for *in vitro* studies are identified and included in the alpha version of the tool.	Project group

**Table 3.** Bias domains with approved definitions in the SEVCO (FEvIR Platform Version 0.80.0, 06.12.2022).

Bias domain	Definition	SEVCO code reference
Selection bias	A bias resulting from methods used to select subjects or data, factors that influence initial study participation, or differences between the study sample and the population of interest	SEVCO:00002
Confounding covariate bias	A situation in which the effect or association between an exposure and an outcome is distorted by another variable. For confounding covariate bias to occur, the distorting variable must be (1) associated with the exposure and the outcome, (2) not in the causal pathway between exposure and outcome and (3) unequally distributed between the groups being compared.	SEVCO:00016
Performance bias	A bias resulting from differences between the received exposure and the intended exposure.	SEVCO:00017
Attrition bias	A bias due to the absence of expected participation or data collection after selection for study inclusion.	SEVCO:00019
Detection bias	A bias due to distortions in any process involved in the determination of the recorded values for a variable.	SEVCO:00020
Analysis bias	A bias related to the analytic process applied to the data.	SEVCO:00021
Reporting bias	A bias due to distortions in the selection or representation of information in study results or research findings.	SEVCO:00023
Early study termination bias	A bias due to the decision to end the study earlier than planned.	SEVCO:00370

**Table 4.** The definition of the codes in the Code Book.

Code category	Code	Definition
Issue	Selection	An issue relating to selection bias
	Confounding	An issue relating to confounding covariates bias
	Performance	An issue relating to performance bias
	Attrition	An issue relating to attrition bias
	Detection	An issue relating to detection bias
	Analysis	An issue relating to analysis bias
	Reporting	An issue relating to reporting bias
	Early termination	An issue relating to early termination bias
	[ad hoc codes]	Ad hoc codes will be created to classify limitations that do not fit into any of the prespecified bias categories (inductive coding)
Time-point	Before exposure	An issue that may affect potential for systematic error prior to the exposure (administration of the chemical substance) in the experiment
	During exposure	An issue that may affect potential for systematic error during the exposure (administration of the chemical substance) in the experiment
	After exposure	An issue that may affect potential for systematic error after the exposure (administration of the chemical substance) in the experiment
Relevance	Higher relevance	Argument or observation that an issue that may affect potential for systematic error is of potentially higher relevance
	Lower relevance	Argument or observation that an issue that may affect potential for systematic error is of potentially lower relevance

**Table 5.** The Code Book.

Level 1	Level 2
Selection	Before exposure
	During exposure
	After exposure
	Higher relevance
	Lower relevance
Confounding	Before exposure
	During exposure
	After exposure
	Higher relevance
	Lower relevance
Performance	Before exposure
	During exposure
	After exposure
	Higher relevance
	Lower relevance
Attrition	Before exposure
	During exposure
	After exposure
	Higher relevance
	Lower relevance
Detection	Before exposure
	During exposure
	After exposure
	Higher relevance
	Lower relevance
Analysis	Before exposure
	During exposure
	After exposure
	Higher relevance
	Lower relevance
Reporting	Before exposure
	During exposure
	After exposure
	Higher relevance
	Lower relevance
Early termination	Before exposure
	During exposure
	After exposure
	Higher relevance
	Lower relevance
[ad hoc codes]	Before exposure
	During exposure
	After exposure
	Higher relevance
	Lower relevance

**Table 6.** An overview of Study 2.

Phase	Task	Responsible
Plan	Define inclusion criteria for Delphi participants (expert panellists).	Project group
	Nominate and recruit expert panellists fulfilling the inclusion criteria. Create the questionnaire addressing the bias domains and items relevant for *in vitro* studies identified in Study 1.	Project group and scientific advisory group
Actions	*Delphi round 1* Expert panellists complete the questionnaire and have the possibility to suggest additional bias domains and items. *Between Delphi rounds 1 and 2* Analyse results from round 1. Feedback from round 1 is given to the expert panellists. Bias domains and items that met criteria for identification of agreement for inclusion in INVITES-IN are removed. Bias domains and items that met criteria for identification of agreement for exclusion from INVITES-IN are removed. New questions may be included, and existing questions may be revised. *Delphi round 2* Feedback from round 1 is given to the expert panellists. Expert panellists complete the questionnaire. Analyse results from round 2.	Project group
	*Workshop* Expert panellists will be guided through a discussion of uncertainties related to bias domains and items for which agreement for inclusion or exclusion is not identified. Prepare transcripts, organise and summarise results.	Project group
Result	Expert agreement on bias domains and items of importance for internal validity of *in vitro* studies is identified.	Project group

**Table 7.** An overview of Study 3.

Phase	Task	Responsible
Plan	Signalling questions are formulated.	Project group
	Guidance for rating the signalling questions is prepared.	
	The process for compiling the results from the rating of the signalling questions into an overall assessment of the risk of bias for each study is created.	
	Invite members of the focus group that interpreted bias domains and items for *in vitro* context (Study 1) to participate in an online workshop.	
Actions	*Workshop* Get feedback on the presentation of and information in the guidance document (Study 1).	Project group
Result	The guidance is revised according to the workshop feedback. The beta version of INVITES-IN is finalised.	Project group

## Abbreviations:

NAM: new approach methodologies
PG: project group
SAG: scientific advisory group

## References

[R1] Alper BS, Dehnbostel J, Afzal M, Subbian V, Soares A, Kunnamo I, Shahin K, and McClure RC. 2021a. “Making Science Computable: Developing Code Systems for Statistics, Study Design, and Risk of Bias.” Journal of Biomedical Informatics 115: 1. 10.1016/j.jbi.2021.103685 PMC9387176 33486066

[R2] Alper BS, Dehnbostel J, Lehmann H, Whaley P, Wilkins KJ, Tufte J, Yurk RA, Ojha N, and Afzal M. 2021b. “For the COVID-19 Knowledge Accelerator (COKA) Initiative.” Scientific Evidence Code System Development Protocol Accessed 16 November 2021, Last revised 8 December 2021). https://docs.google.com/document/d/1pzGLdyVCKcu3s2gfSfPpXDQLlQsFnLZR14ldw0nD1g0.

[R3] Boutron I, Page MJ, Higgins JPT, Altman DG, Lundh A, and Hróbjartsson A. 2022. “Chapter 7: Considering Bias and Conflicts of Interest among the Included Studies.” In Cochrane Handbook for Systematic Reviews of Interventions Version 6.3 (Updated February 2022), edited by Higgins JPT, Thomas J, Chandler J, Cumpston M, Li T, Page MJ, and Welch VA. Cochrane. https://training.cochrane.org/handbook/current/chapter-07.

[R4] Cochrane Collaboration. 2005. “Glossary of Terms in the Cochrane Collaboration,” Version 4.2.5. http://aaz.hr/resources/pages/57/7.%20Cochrane%20glossary.pdf.

[R5] Cooper GS, Lunn RM, Ågerstrand M, Glenn BS, Kraft AD, Luke AM, and Ratcliffe JM. 2016. “Study Sensitivity: Evaluating the Ability to Detect Effects in Systematic Reviews of Chemical Exposures.” Environment International 92–93: 605–16. 10.1016/j.envint.2016.03.017 PMC5110036 27156196

[R6] Dalkey N, and Helmer O. 1963. “An Experimental Application of the DELPHI Method to the Use of Experts.” Management Science 9 (3): 458–467. 10.1287/mnsc.9.3.458

[R7] Dent M, Amaral RT, Da Silva PA, Ansell J, Boisleve F, Hatao M, Hirose A, 2018. “Principles Underpinning the Use of New Methodologies in the Risk Assessment of Cosmetic Ingredients.” Computational Toxicology 7: 20–26. 10.1016/j.comtox.2018.06.001

[R8] Dirven H, Vist GE, Bandhakavi S, Mehta J, Fitch SE, Pound P, Ram R, 2021. “Performance of Preclinical Models in Predicting Drug-Induced Liver Injury in Humans: A Systematic Review.” Scientific Reports 11 (1): 6403. 10.1038/s41598-021-85708-2 33737635 PMC7973584

[R9] ECHA. 2016. “New Approach Methodologies in Regulatory Science.” In Proceedings of a Scientific Workshop: Helsinki, 19–20 April 2016 European Chemicals Agency. https://echa.europa.eu/documents/10162/21838212/scientific_ws_proceedings_en.pdf/a2087434-0407-4705-9057-95d9c2c2cc57.

[R10] ECHA. 2023. “In Vitro Methods.” European Chemicals Agency https://echa.europa.eu/support/registration/how-to-avoid-unnecessary-testing-on-animals/in-vitro-methods.

[R11] Eick SM, Goin DE, Chartres N, Lam J, and Woodruff TJ. 2020. “Assessing Risk of Bias in Human Environmental Epidemiology Studies Using Three Tools: Different Conclusions from Different Tools.” Systematic Reviews 9 (1): 249. 10.1186/s13643-020-01490-8 33121530 PMC7596989

[R12] EPA. 2018. “Strategic Plan to Promote the Development and Implementation of Alternative Test Methods within the TSCA Program.” EPA-740-R1–8004. United States Environmental Protection Agency https://www.epa.gov/sites/default/files/2018-06/documents/epa_alt_strat_plan_6-20-18_clean_final.pdf.

[R13] EPA. 2022. ORD Staff Handbook for Developing IRIS Assessments Washington, DC: U.S. EPA Office of Research and Development (EPA/600/R-22/268). https://ordspub.epa.gov/ords/eims/eimscomm.getfile?p_download_id=545991.

[R14] EPA. 2023. “Draft Protocol for Systematic Review in TSCA Risk Evaluations.” United States Environmental Protection Agency Accessed 10 February 2023. https://www.epa.gov/assessing-and-managing-chemicals-under-tsca/draft-protocol-systematic-review-tsca-risk-evaluations.

[R15] FEvIR Platform Version 0.80.0. (06.12.2022) Scientific Evidence Code System (SEVCO) Computable Publishing^®^: CodeSystem Viewer. https://fevir.net/resources/CodeSystem/27270#SEVCO:00001.

[R16] Frampton G, Whaley P, Bennett M, Bilotta G, Dorne J-LCM, Eales J, James K, 2022. “Principles and Framework for Assessing the Risk of Bias for Studies Included in Comparative Quantitative Environmental Systematic Reviews.” Environmental Evidence 11 (1): 12. 10.1186/s13750-022-00264-0 PMC10805236 38264537

[R17] Gilmour N, Kern PS, Alépée N, Boislève F, Bury D, Clouet E, Hirota M, 2020. “Development of a Next Generation Risk Assessment Framework for the Evaluation of Skin Sensitisation of Cosmetic Ingredients.” Regulatory Toxicology and Pharmacology 116: 104721. 10.1016/j.yrtph.2020.104721 32645429

[R18] Krueger RA, Casey MA, Donner J, Kirsch S, and Maack JN. 2001. “Designing and Conducting Focus Group Interviews, Social Development Papers 31.” In Social Analysis: Selected Tools and Techniques Washington, DC: World Bank. https://documents1.worldbank.org/curated/en/568611468763498929/pdf/282790SDP136.pdf.

[R19] Momen NC, Streicher KN, da Silva DTC, Descatha A, Frings-Dresen MHW, Gagliardi D, Godderis L, 2022. “Assessor Burden, Inter-Rater Agreement and User Experience of the RoB-SPEO Tool for Assessing Risk of Bias in Studies Estimating Prevalence of Exposure to Occupational Risk Factors: An Analysis from the WHO/ILO Joint Estimates of the Work-Related Burden of Disease and Injury.” Environment International 158: 107005. 10.1016/j.envint.2021.107005 34991265 PMC8685606

[R20] NTP OHAT. 2015. “Protocol to Evaluate the Evidence for an Association between Perfluorooctanic Acid (PFOA) and Perfluorooctane Sulfonate (PFOS) Exposure and Immunotoxicity,” 65–70. Office of Health Assessment and Translation (OHAT), Division of the National Toxicology Program, National Institute of Environmental Health Sciences https://ntp.niehs.nih.gov/ntp/ohat/pfoa_pfos/protocol_201506_508.pdf.

[R21] NTP OHAT. 2019. “Handbook for Conducting a Literature Based Health Assessment Using OHAT Approach for Systematic Review and Evidence Integration.” Office of Health Assessment and Translation (OHAT), Division of the National Toxicology Program, National Institute of Environmental Health Sciences https://ntp.niehs.nih.gov/ntp/ohat/pubs/handbookmarch2019_508.pdf.

[R22] PARC. 2023. “PARC_What we do_Thematic areas_News_Events_Scientific Publications” Accessed 11 May 2023. https://www.eu-parc.eu/#news-and-events.

[R23] Roth N, Zilliacus J, and Beronius A. 2021. “Development of the SciRAP Approach for Evaluating the Reliability and Relevance of in Vitro Toxicity Data.” Frontiers in Toxicology 3: 746430. 10.3389/ftox.2021.746430 35295161 PMC8915875

[R24] Savović J, Jones HE, Altman DG, Harris RJ, Jüni P, Pildal J, Als-Nielsen B, 2012. “Influence of Reported Study Design Characteristics on Intervention Effect Estimates from Randomized, Controlled Trials.” Annals of Internal Medicine 157 (6): 429–438. 10.7326/0003-4819-157-6-201209180-00537 22945832

[R25] Sterne JAC, Hernán MA, Reeves BC, Savović J, Berkman ND, Viswanathan M, Henry D, 2016. “ROBINS-I: A Tool for Assessing Risk of Bias in Non-Randomised Studies of Interventions.” BMJ 355: i4919. 10.1136/bmj.i4919 27733354 PMC5062054

[R26] Sterne JAC, Page MJ, Savović J, Elbers RG, Blencowe NS, Boutron I, Cates CJ, 2019. “RoB 2: A Revised Tool for Assessing Risk of Bias in Randomised Trials.” BMJ 366: l4898. 10.1136/bmj.l4898 31462531

[R27] EFSA, Tarazona J, Kass G, Dorne J-L, Liem D, Paraskevopoulos K, Kleiner J, Heppner C, and Hugas M. 2022. “Theme (Concept) Paper – New Approach Methodologies.” EFSA Supporting Publications 19 (5): E200502E. 10.2903/sp.efsa.2022.e200502

[R28] Tran L, Tam DNH, Elshafay A, Dang T, Hirayama K, and Huy NT. 2021. “Quality Assessment Tools Used in Systematic Reviews of in Vitro Studies: A Systematic Review.” BMC Medical Research Methodology 21 (1): 101. 10.1186/s12874-021-01295-w 33964880 PMC8106836

[R29] Verhagen AP, de Vet HC, de Bie RA, Kessels AG, Boers M, Bouter LM, and Knipschild PG. 1998. “The Delphi List: A Criteria List for Quality Assessment of Randomized Clinical Trials for Conducting Systematic Reviews Developed by Delphi Consensus.” Journal of Clinical Epidemiology 51 (12): 1235–1241. 10.1016/s0895-4356(98)00131-0 10086815

[R30] VKM. 2023. “The Norwegian Scientific Committee of Food and Environment (VKM) Participates in the European Partnership for the Assessment of Risks from Chemicals (PARC)” Accessed 11 May 2023. https://vkm.no/english/parc/parceuropeanpartnershipfortheassessmentofrisksfromchemicals.4.7205492a1864a8c8da2dcfd9.html.

[R31] Whaley P, Hooijmans CR, and Wattam S. (in preparation). “Literature-Based Discovery of Assessment Criteria for in Vitro Studies: A Method and Item Bank” 10.1093/toxsci/kfae083 PMC11424884 38964352

[R32] Whiting PF, Wolff R, Mallett S, Simera I, and Savović J. 2017. “A Proposed Framework for Developing Quality Assessment Tools.” Systematic Reviews 6 (1): 204. 10.1186/s13643-017-0604-6 29041953 PMC5646161

